# Inhibition of HCV translation by disrupting the structure and interactions of the viral CRE and 3′ X-tail

**DOI:** 10.1093/nar/gkv142

**Published:** 2015-02-20

**Authors:** Andrew Tuplin, Madeleine Struthers, Jonathan Cook, Kirsten Bentley, David J. Evans

**Affiliations:** 1 School of Molecular and Cellular Biology, Faculty of Biological Sciences, University of Leeds, Leeds LS2 9JT, UK; 2 School of Life Sciences, University of Warwick, Gibbet Hill Road, Coventry CV4 7AL, UK

## Abstract

A phylogenetically conserved RNA structure within the NS5B coding region of hepatitis C virus functions as a *cis*-replicating element (CRE). Integrity of this CRE, designated SL9266 (alternatively 5BSL3.2), is critical for genome replication. SL9266 forms the core of an extended pseudoknot, designated SL9266/PK, involving long distance RNA–RNA interactions between unpaired loops of SL9266 and distal regions of the genome. Previous studies demonstrated that SL9266/PK is dynamic, with ‘open’ and ‘closed’ conformations predicted to have distinct functions during virus replication. Using a combination of site-directed mutagenesis and locked nucleic acids (LNA) complementary to defined domains of SL9266 and its interacting regions, we have explored the influence of this structure on genome translation and replication. We demonstrate that LNAs which block formation of the closed conformation inhibit genome translation. Inhibition was at least partly independent of the initiation mechanism, whether driven by homologous or heterologous internal ribosome entry sites or from a capped message. Provision of SL9266/PK *in trans* relieved translational inhibition, and mutational analysis implied a mechanism in which the closed conformation recruits a cellular factor that would otherwise suppresses translation. We propose that SL9266/PK functions as a temporal switch, modulating the mutually incompatible processes of translation and replication.

## INTRODUCTION

**Figure 1.**
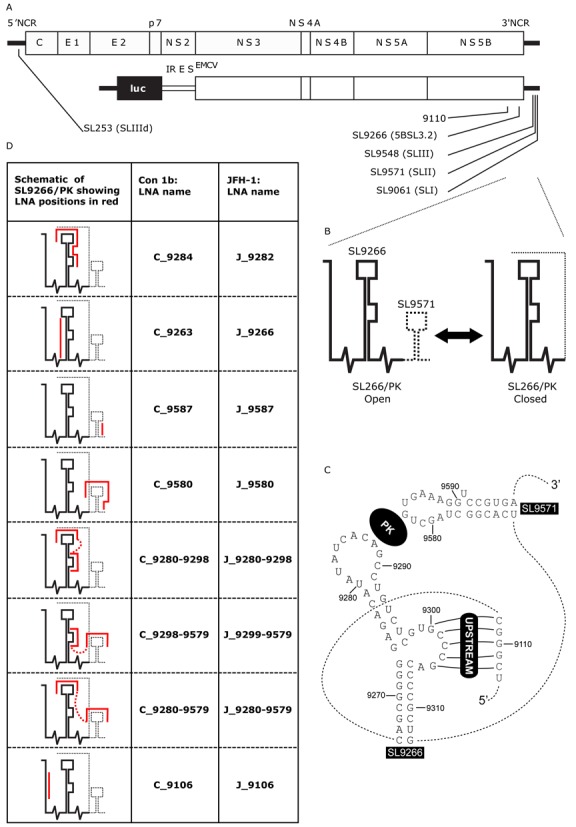
(**A**) Schematic diagram of the genome of HCV JFH-1 (top) and Con1b-luc-rep (below) indicating the location of RNA stem–loop structures (SL) using both standardised positional references and naming schemes from previous publications. (**B**) Schematic representation of the dynamic SL9266/PK pseudoknot showing its open and closed conformations. A dashed line represents genome regions that switch between alternative conformations. (**C**) Schematic of representation of SL9266 and SL9571 in Con1b showing individual nucleotides labelled with standardised positional references and locations of the kissing loop (PK) and upstream interactions labelled in reverse camera. Dashed lines represents sequence regions not shown. (**D**) Table shows schematic representations of SL9266/PK with antisense-LNA binding sites represented by red lines, non-specific linkers are shown as dashed red lines. LNA-oligonucleotides are named for the upstream nucleotide to which they are predicted to bind and are preceded by a C (for Con1b) or J (for JFH-1)—depending on which HCV isolate they target.

Hepatitis C virus (HCV) is member of the *Flaviviridae* family, within the *Hepacivirus* genus, and is a major cause of liver disease, estimated to infect over 170 million people worldwide (1). The virus possesses a positive-sense, single-stranded RNA genome ∼9.6 kb in length encoding a single open reading frame (ORF) flanked by 5′ and 3′ non-coding regions (NCRs) (Figure 1A). The 5′NCR and adjacent ORF incorporates an internal ribosome entry site (IRES; nucleotides 39–371) essential for translation initiation by direct recruitment of the 40S ribosomal subunit to the AUG start codon (2–5). HCV RNA genomes are multifunctional molecules, acting as a template for both translation and replication. The initial translation events are essential for subsequent replication, through the production of non-structural proteins including the RNA-dependent RNA polymerase (RdRp). Following early translation events HCV genomic RNA is used as a template to generate a double-stranded replication intermediate, the negative-strand of which acts as a template for production of further positive-sense daughter molecules. Viruses, including presumably HCV, have evolved many ways to control the key, yet mutually exclusive, processes of translation and replication within their life cycle. These include a variety of feedback loops dependent upon interaction of viral or cellular proteins with sequence motifs, or more often secondary structures, within the virus genome (6–8).

The HCV genome carries at least two distinct types of RNA structure. The first, designated genome-scale ordered RNA structure (GORS), is generally not phylogenetically conserved (though the presence of structure is) and extends throughout the genome (9). GORS is predicted to be involved in subversion or evasion of the innate immune response (10,11). In addition, the HCV genome contains a number of discrete and highly conserved RNA structures, primarily located in the 5′ and 3′NCRs and extending into the adjacent coding region. Within the 5′NCR the IRES consists of three RNA stem–loops (domains II, III and IV) and a pseudoknot (at the base of stem III), essential for ribosome recruitment and translation initiation (12–15). A number of stem–loops within both the 5′NCR (domains I and II) and the start of the ORF (designated SLV/SL47 and SLVI/SL87 (16–18)), are also required for efficient genome replication (19,20).

The 3′NCR has roles in both initiation of anti-genome synthesis and influencing IRES-mediated translation (21,22). It is composed of three distinct domains. A hypervariable region directly downstream of the ORF followed by a poly U/UC stretch of variable length and a highly conserved domain at the 3′ terminus designated the X-tail. The latter comprises three RNA stem–loops numbered SL9548, SL9571 and SL9061 (23) (alternatively designated SLI, SLII and SLIII respectively (24)). Within the adjacent NS5B coding region there are at least five additional phylogenetically conserved stems loops designated SL9033, SL9132, SL9217, SL9266 and SL9324 (17,18). Of these, SL9266 (alternatively termed 5BSL3.2 (25) or SL-V (26)) consists of a 12 nt terminal loop presented on upper and lower hetero-duplexed domains with a sub-terminal 8 nt. bulge loop interrupting the 3′ proximal stem (Figure 1A).

It has been demonstrated, in both sub-genomic replicon and full length replicating virus systems, that disrupting base pairing within the duplexed stems of SL9266 prevents or severely inhibits HCV replication (25,26). Consequently, SL9266 is a *cis*-acting replicating element (CRE). SL9266 does not act in isolation but as the core of a complex tertiary structure, involving long-range RNA–RNA interactions, essential for efficient virus replication. The 8 nt bulge loop has been predicted to form several long distance RNA–RNA interactions, with genetic or biophysical evidence for binding to complementary sequences in an upstream coding region centred on nt 9110 (23,27) or to the single-stranded terminal loop of the IRES IIId domain (28,29). The terminal loop of the SL9266 forms a tertiary ‘kissing loop’ interaction with the terminal loop region of SL9571 in the X-tail (30). SL9266 therefore forms the core of an extended pseudoknot which we earlier designated SL9266/PK (23). Additional studies suggest that these terminal and bulge loop tertiary interactions are structurally independent and that binding of one does not influence formation of the other (23,31).

We previously published reverse genetic and biochemical structural data showing that the interactions involved in the formation of SL9266/PK are dynamic (23). A combination of biochemical mapping using SHAPE (selective 2′-hydroxyl acylation analysed by primer extension) and phenotypic analysis of mutants, in both sub-genomic replicon and full length replicating virus systems, revealed that SL9266/PK forms alternative closed and open conformations (Figure 1B), both of which are required for efficient completion of the virus replication cycle. In the closed conformation the ‘kissing loop’ interaction—between the terminal loop of SL9266 and the sequences that occupy the terminal loop of SL9571—prevents the formation of the duplexed stem region of SL9571. Conversely, in the open conformation the ‘kissing loop’ interaction is absent, the terminal loops of both SL9266 and SL9571 are single stranded and the duplexed stem of SL9571 can and does form. SHAPE analysis of mutated molecules suggested that the thermodynamic equilibrium between the two alternative conformations favoured the open conformation in the genotype 1 Con1b and H77 isolates and the closed conformation in genotype 2a JFH-1.

Here, we present further supporting evidence that SL9266/PK functions as a dynamic riboswitch with open and closed conformations. We demonstrate that translation from the HCV IRES is specifically enhanced by the closed conformation while the open conformation is associated with reduced translation levels. We propose that SL9266/PK functions as an essential temporal switch, in which the open and closed conformations modulate the mutually incompatible translation and replication events critical for generation of progeny. These studies form the basis for an improved understanding of HCV replication and further define means to inhibit the function of a phylogenetically conserved potential target for therapeutic intervention.

## MATERIALS AND METHODS

### RNA stem–loop nomenclature

RNA stem–loops are designated according to the published nomenclature of Kuiken *et al*. (32), essentially by the position of the first 5′ paired nucleotide in the structure aligned and referenced to the H77 complete genome sequence (GenBank accession #AF011753) (23). Of relevance to this report, stem–loop structures named 5BSL3.1–3.3, SLVI–IV or SL9011, SL9061 and SL9118 are designated here SL9217, SL9266 and SL9324 respectively. Likewise, the three structures that together form the X-tail (SLIII, SLII and SLI) are designated SL9548, SL9571 and SL9601 respectively. The higher order structure formed by the ‘kissing loop’ interaction between the terminal loops of SL9266 and SL9571 has been designated SL9266/PK (23).

### Design and nomenclature of locked nucleic acid (LNA) antisense oligonucleotides

Antisense-LNA oligonucleotides (hereafter antisense-LNAs; Exiqon) were designed to be complementary to target sequences within genotype 1b (Con1b) or genotype 2a (JFH-1) SL9266/PK sequences (Figure 1C and Supplementary data S1). Individual antisense-LNAs were either complementary to specific subunit motifs of SL9266/PK or to spatially separate structural motifs within different subunits of the higher order structure—in which case antisense motifs were separated by non-specific linker sequences. Antisense-LNAs were numbered for the position of the 5′ nucleotide (sense strand) of the target motif/s. They were designed with a minimum of three 5′ and 3′ terminal LNA nucleotides, with non-LNA nucleotides limited to four base stretches and with similar thermodynamic and sequence motif binding properties (LNA design tools, Exiqon). The relative binding efficiency of antisense-LNAs and their complementary RNA sequences was biochemically quantified by gel shift assays (Supplementary data S2).

### HCV cDNA plasmids, reporter construction and mutagenesis

The parental firefly luciferase-encoding Con1b sub-genomic replicon pFK5.1 (termed here Con1b-luc-rep) has been described previously (33) (Figure 1A). The HCVcc—designated pFK-J6/JFH-1-C-846 (for convenience termed here as J6/JFH-1)—was generously provided by Takaji Wakita and NIH and has previously been fully described (34) (Figure 1A). Replication-incompetent derivatives of Con1b-luc-rep were generated by a GDD>GND substitution, within the active site of the NS5B polymerase as described previously (27). *Renilla* luciferase RNA was generated from the cDNA plasmid pRL (Promega).

A translation-only reporter construct for genotype 1b (designated Con1b_luc_trans; Figure 3) was constructed by overlap extension PCR of the Con1b-luc-rep cDNA template. A fragment spanning the complete 5′NCR to the end of firefly luciferase was amplified by PCR and joined to a second PCR amplification product spanning the complete NS5B and 3′NCR domains by overlap extension PCR. The final overlap PCR product incorporated in sequential order from the 5′ end; a unique Sac I restriction site, hammerhead ribozyme sequence (which cleaves transcribed RNA immediately upstream of the nucleotide 1 of the 5′NCR), complete Con1b 5′NCR and the first 48 nts of the core coding region in frame with firefly luciferase, an EMCV IRES (encephalomyocarditis virus internal ribosome entry site) immediately upstream of an AUG start codon, V5 peptide tag fused in frame to the Con1b NS5B coding region, complete 3′NCR and a unique Spe I restriction site.

**Figure 2.**
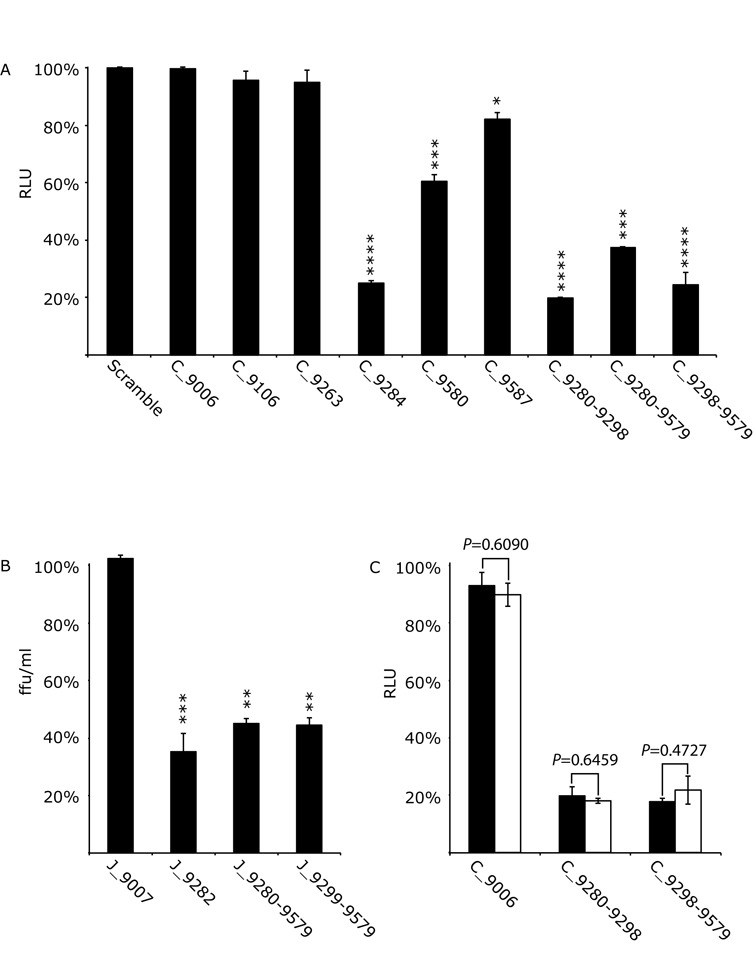
The effect of SL9266/PK antisense-LNAs on replication and translation in Huh 7.5 cells of the Con1b-luc-rep sub-genomic replicon and replication of J6/JFH-1 virus. Results represent the average of at least three independent assays, and are expressed as a percentage of control transfections/infections lacking antisense-LNAs or scrambled LNA-oligonucleotides (error bars indicate the standard error from the mean and stars the degrees of significance from untreated controls). (**A**) Con1b-luc-rep: Relative luciferase levels (Firefly/Renilla) were measured 24 h post-RNA transfection. (**B**) J6/JFH-1 HCVcc: Replication was measured as focus forming units/ml (ffu/ml) 24 h post-infection in the presence of antisense-LNAs. (**C**) Relative luciferase levels (Firefly/Renilla) from transfected Con1b-luc-rep RNA compared to a replication defective mutant bearing a GDD>GND mutation within the active site of RdRp. Relative luciferase levels were measured 6 h post-transfection and expressed as a percentage of control transfections for either the wild type (filled black bars) or GND mutant (open bars). Significance between corresponding wild type and GND mutants indicated.

**Figure 3.**
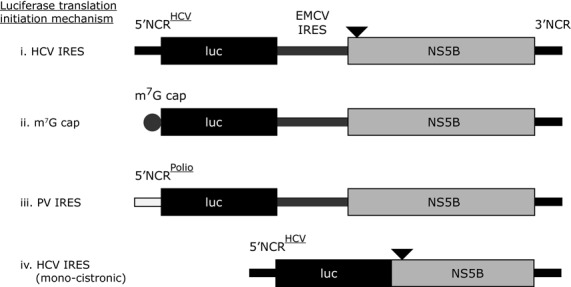
Schematic diagrams of translation only reporter constructs. (**i**) Represents a bicistronic construct with the 5′NCR from HCV upstream of the first 66 nucleotides of the HCV core coding region fused in frame to firefly luciferase. A downstream EMCV IRES initiates translation from the NS5B coding region, which is upstream of the complete HCV 3′NCR (in ΔNS5B versions NS5B is not translated due to a stop codon incorporated at the third codon position, which is represented by the black triangle). Con1b and JFH-1 versions of this reporter were constructed and assayed. The Con1b reporter construct was further modified by replacing the 5′NCR with either an m^7^G cap (**ii**), a polio virus 5′NCR (**iii**) or by deleting the EMCV IRES and upstream portion of NS5B (an in frame stop codon was incorporated into the 3′ truncated NS5B, represented by a black filled triangle) (**iv**).

An alternative version of Con1b_luc_trans was made, deficient in NS5B synthesis (designated Con1b_luc_trans:ΔNS5B; Figure 3), in which the third codon position of the V5 sequence was mutated to a stop codon (A_2683_T) to prevent translation of the tagged NS5B coding region. Mutations were also made to remove an in-frame AUG at codon 2 within the NS5B coding region (A_2725_G+T_2726_C). Premature termination of NS5B translation was confirmed by western blotting after *in vitro* and *in vivo* translation (in Huh 7.5 cells and rabbit reticulate lysate respectively) and compared to active NS5B translation from a wild-type transcript (data not shown). The overlap PCR generated fragment was incorporated into pBluescript II KS (−) (Stratagene) downstream of a bacteriophage T7 transcription promoter sequence (T7 sequence) between unique Sac I and Spe I restriction sites.

Two plasmids for constructing the JFH-1 based translation-only reporter cDNA constructs were synthesized by GeneArt (Life Technologies). The first carried a unique Sac I restriction site, a 5′ hammerhead ribozyme sequence, the complete JFH-1 5′NCR with the first 48 nts of the core coding region and 63 nts of firefly luciferase. The second plasmid contained the final 433 nts of the EMCV IRES, a V5 peptide tag sequence, the complete NS5B coding region and 3′NCR of HCV JFH-1 followed by a unique *Spe* I restriction site. These two plasmids were used to construct a JFH-1-based equivalent of Con1b_luc_trans using standard cloning techniques with the final bicistronic plasmid being designated JFH-1_luc_trans (Figure 3). As before, an alternative version deficient in NS5B translation was also constructed and designated JFH-1_luc_trans:ΔNS5B.

The HCV 5′NCR in Con1b_luc_trans:ΔNS5B was replaced with the complete 5′NCR sequence of poliovirus (type 3 Leon GenBank accession no. X00596.1), creating Polio_luc_trans:ΔNS5B (Figure 3). A cDNA insert was generated by overlap PCR incorporating in sequential order a unique Sac I restriction site, the complete poliovirus 5′NCR and the 5′ 1159 nts of firefly luciferase. This fragment was cloned into and replaced the equivalent regions in Con1b_luc_trans:ΔNS5B between the Sac I and Xba I restriction sites; the 3′ NS5B coding region and 3′NCR from Con1b remaining unchanged. A mono-cistronic translation reporter lacking the EMCV IRES, V5 peptide sequence tag and 5′ 321 nts of the NS5B encoding region was built (designated Con1b_luc_trans:ΔEMCV (Figure 3), by deleting the fragment between unique restriction sites Eag I and Zra I. Con1b_luc_trans: Δ5′NCR+ΔNS5B cDNA (Figure 3) was transcribed from a Con1b_luc_trans: ΔNS5B PCR product template, initiating at the 5′ nucleotide position of firefly luciferase and terminated at the 3′ end of the 3′NCR. Mutations were introduced using the Stratagene QuikChange™ system according to the manufacturers instructions, their presence confirmed by DNA sequencing, and rebuilt into the parental plasmid between unique restriction sites.

### *In vitro* RNA transcription

One microgram of template linearized with an appropriate enzyme (Sca I for Con1b_luc_trans/:ΔNS5B, Polio_luc_trans:ΔNS5B and Con1b_luc_trans:ΔEMCV+:ΔNS5B, BspH I for JFH-1_luc_trans:ΔNS5B and Xba I for pRL) or terminated with a 3′ *cis*-acting ribozyme (J6/JFH-1 plasmid) was used to prime a T7 MEGAscript kit (Life Technologies), and used according to the manufacturers instructions. PCR products amplified with a sense primer containing a T7 promoter were generated as templates for transcription of wild type and mutant NS5B-3′NCR RNA for *trans* supplementation assays. Con1b_luc_trans:Δ5′NCR RNA was generated from a PCR amplified template as described earlier, 250 ng was used as template for *in vitro* production of 5′ [m7G(5′)ppp(5′)G] capped (m^7^G capped) RNA using a T7 mMessage mMachine kit (Life Technologies) according to the manufactures instructions. Following transcription, residual DNA template was removed by DNase 1 (Life Technologies) treatment and RNA purified with an RNeasy mini-kit column (Qiagen). RNA integrity was confirmed by denaturing agarose gel electrophoresis and quantified by NanoDrop spectroscopy.

### Cell culture and transfections

Monolayers of the human hepatoma cell line Huh 7.5 (a generous gift from Charles Rice) were maintained in Dulbecco's modified minimal essential medium (DMEM) supplemented with 10% (v/v) foetal bovine serum (FBS) (Life Technologies), 1% non-essential amino acids, 2 mM l-glutamine and 100 U penicillin/100 μg streptomycin/ml (DMEM P/S). Cells were passaged after trypsin/EDTA treatment, seeded at dilutions of 1:3 to 1:5 and maintained at 37°C in 5% CO_2_.

### Reporter transfection and analysis

Huh 7.5 cells were seeded in 24-well plates at ∼3 × 10^5^ cells/well and maintained overnight in DMEM P/S before RNA transfection using Lipofectamine 2000 (Life Technologies). Briefly, monolayers at ∼90% confluence were washed twice in phosphate buffered saline (PBS) before adding 500 μl of DMEM supplemented with 1% non-essential amino acids and 2 mM l-glutamine before addition of 100 μl of transfection medium in a dropwise manner. Transfection medium was prepared according to the manufacturer's instructions with 2 μl Lipofectamine 2000, 0.32 pmol of reporter RNA, 0.32 pmoles of renilla luciferase RNA and made up to 100 μl with Opti-Mem reduced-serum media (Life Technologies). Where appropriate, antisense-LNA, scrambled LNA oligonucleotide or *in vitro* synthesized SL9266/PK RNA, was included in the transfection medium. After transfection, monolayers were maintained for 6 h before being washed twice with PBS, lysed with 0.5 ml Glo-Lysis Buffer (Promega) and stored frozen prior to analysis using Dual-luciferase substrate (Promega) and a Turner TL-20 luminometer. In the case of Con1b-luc-rep, media was changed after 4 h, monolayers washed twice with PBS and replaced with 1 ml of DMEM P/S. They were then maintained for 24 h before harvesting and analysed as described earlier.

### Virus analysis and quantification

Huh 7.5 cells were seeded in a 24-well plate at ∼3 × 10^5^ cells/well and maintained overnight in DMEM P/S. The following day monolayers were washed twice with PBS and transfected with 300 nmol of antisense-LNA using Lipofectamine 2000 as described earlier. Four hours post-transfection monolayers were washed twice with PBS and once with DMEM P/S before incubating for 2 h with 300 μl of filtered J6/JFH-1 virus supernatant (2 × 10^2^ ffu/ml). Virus media was then removed, monolayers washed twice with PBS and replaced with 1 ml DMEM P/S. Twenty four hours after infection, monolayers were washed twice with PBS, fixed with 1 ml 4% paraformaldehyde for 20 min, and washed twice in PBS before permeabilization with 0.1% Triton PBS for 7 min with constant agitation. After a subsequent PBS wash infected cells were detected using a polyclonal sheep antibody to NS5A (αNS5A; generously supplied by Mark Harris) diluted 1:5000 in 10% FBS. After incubation for 1 h. primary antibody was detected using an AlexaFluor594-conjugated secondary anti-sheep antibody (1:500 in 10% FBS; Invitrogen), washed in PBS and stored under PBS containing 0.1% VECTASHIELD DAPI (Vector Laboratories) before analysis by UV microscopy. Infected foci were counted and expressed in focus forming units per ml (ffu/ml).

### Statistical analysis

Statistical analysis was carried out using two-tailed Student's *t*-tests for unpaired samples of equal variance. *P* values of ≤0.05 (*), ≤0.01 (**), ≤0.001 (***) and ≤0.0001 (****) were considered to represent degrees of significance.

## RESULTS

Reverse genetic analysis has demonstrated that deletion or mutation of base-pairing components within SL9266/PK inhibit virus replication to varying degrees (23,27,30,35). This effect has been observed for different genotypes and in different assay systems, including sub-genomic replicons and full-length virus (HCVcc), in both cell culture and animal model systems. This phenotype may result from the direct inhibition of replication *per se* or indirectly by the inhibition of translation, resulting in the subsequent suppression of replication. To investigate this further we studied the inhibitory influence of degradation resistant LNA oligonucleotides complementary to features of SL9266/PK on virus replication and translation, reasoning that a bound LNA would interrupt the function of SL9266/PK. A range of antisense-LNAs were designed to inhibit formation of the pseudoknot, to simultaneously anneal to the upstream and downstream components of SL9266/PK—mimicking a locked closed conformation—or to block different potential sequence specific signal motifs (Figure 1C).

### Steric hindrance of SL9266/PK: replicon and HCVcc phenotypes

The effect of SL9266/PK antisense-LNAs on HCV replication was assayed in Huh 7.5 cells using a Con1b sub-genomic replicon system 24 h post-transfection and expressed as a function of relative luciferase translation compared to control assays lacking LNA-oligonucleotides (Figure 2A). Scrambled LNA-oligonucleotides or antisense-LNAs targeting an unstructured region within the HCV genome (C_9006) (17), upstream of SL9266/PK, were assayed with no difference observed from untreated control assays. Antisense-LNAs specific to SL9266 domains partially or completely involved in pseudoknot formation (LNAs C_9284 and C_9280–9298; Figure 1C) suppressed replication by ∼75% and ∼80% respectively (*P* ≤ 0.0001). Specifically targeting components of SL9571 (LNAs C_9587 and C_9580) resulted in less—although still statistically significant – repression of HCV replication. Antisense-LNAs simultaneously targeting either the terminal or bulge loops SL9266 and the terminal loop of SL9571 (LNAs C_9280-9579 and C_9298-9579) inhibited replication by ∼63% (*P* ≤ 0.001) and 75% respectively (*P* ≤ 0.0001). In contrast, antisense-LNAs designed to only block SL9266 duplex-stem formation (LNA C_9263) or to bind to the upstream region 9110, with which the bulge loop of SL9266 interacts (LNA C_9106), had no statistically significant effect on replication.

The structural dynamics of SL9266/PK differ between the predominant HCV model systems (Con1b/H77 and JFH-1) (23). To investigate whether there were also differences between the inhibitory effects of pseudoknot-specific LNAs we additionally tested a subset of antisense-LNAs specific for JFH-1 SL9266/PK. Their effect on full-length J6/JFH-1 virus replication in the HCVcc system was assayed by infection of LNA transfected cells, measured by immunofluorescence 24 h post-infection and expressed in terms of focus-forming units per ml (ffu/ml) compared to controls lacking LNA-oligonucleotides (Figure 2B). Targeting the pseudoknot with antisense-LNAs designed to the single-stranded loops and upper duplex of SL9266 (J_9282) inhibited virus replication by ∼65% (*P* = 0.0011). Single antisense-LNAs simultaneously targeting either the terminal loop regions of SL9266 and SL9571 (J_9280–9579) or the bulge loop of SL9266 and terminal loop region of SL9571 (J_9299–9579) both inhibited virus replication by ∼55% (*P* = 0.0032 and 0.0031 respectively). As demonstrated in the genotype 1b system, an antisense-LNA, complementary to an unstructured region of the virus genome (J_9007), had no effect in virus replication.

These assays demonstrate that complementary oligonucleotides targeting the phylogenetically conserved SL9266/PK pseudoknot can inhibit genome replication. All antisense-LNAs were demonstrated to hybridize to template molecules (Supplementary data S2) and no correlation was observed between measured or predicted annealing efficiencies and levels of inhibition. The absence of inhibition—for example by C_9006—implies that the inhibitory effect is not mediated by a simple block to ribosome progression, or by an RNAi-type degradation of the virus genome.

### Steric hindrance of SL9266/PK: translation reporter phenotype

Having demonstrated that targeting SL9266/PK blocks HCV replication the impact on genome translation was assayed (Figure 2C). Using a replication-defective sub-genomic replicon bearing a GDD>GND substitution in the NS5B polymerase active site, we investigated the influence on translation of a subset of LNAs earlier demonstrated to have the greatest inhibitory effect on Con1b replication. The effect of antisense-LNAs C_9280-9298 and C_9298-9579 on luciferase translation from the GND mutant, relative to controls lacking LNA-oligonucleotides, was measured 6 h post-transfection and compared to an equivalent parallel assay using replication competent replicons. Antisense-LNAs complementary to SL9266/PK inhibited luciferase translation from the GND mutant, which was unaffected by the C_9006 control. Relative levels of luciferase inhibition from the GND mutant were indistinguishable from those observed for the parental (GDD) replicon treated with the same oligonucleotides, suggesting that SL9266/PK antisense-LNAs were specifically inhibiting HCV translation rather than genome replication.

**Figure 4.**
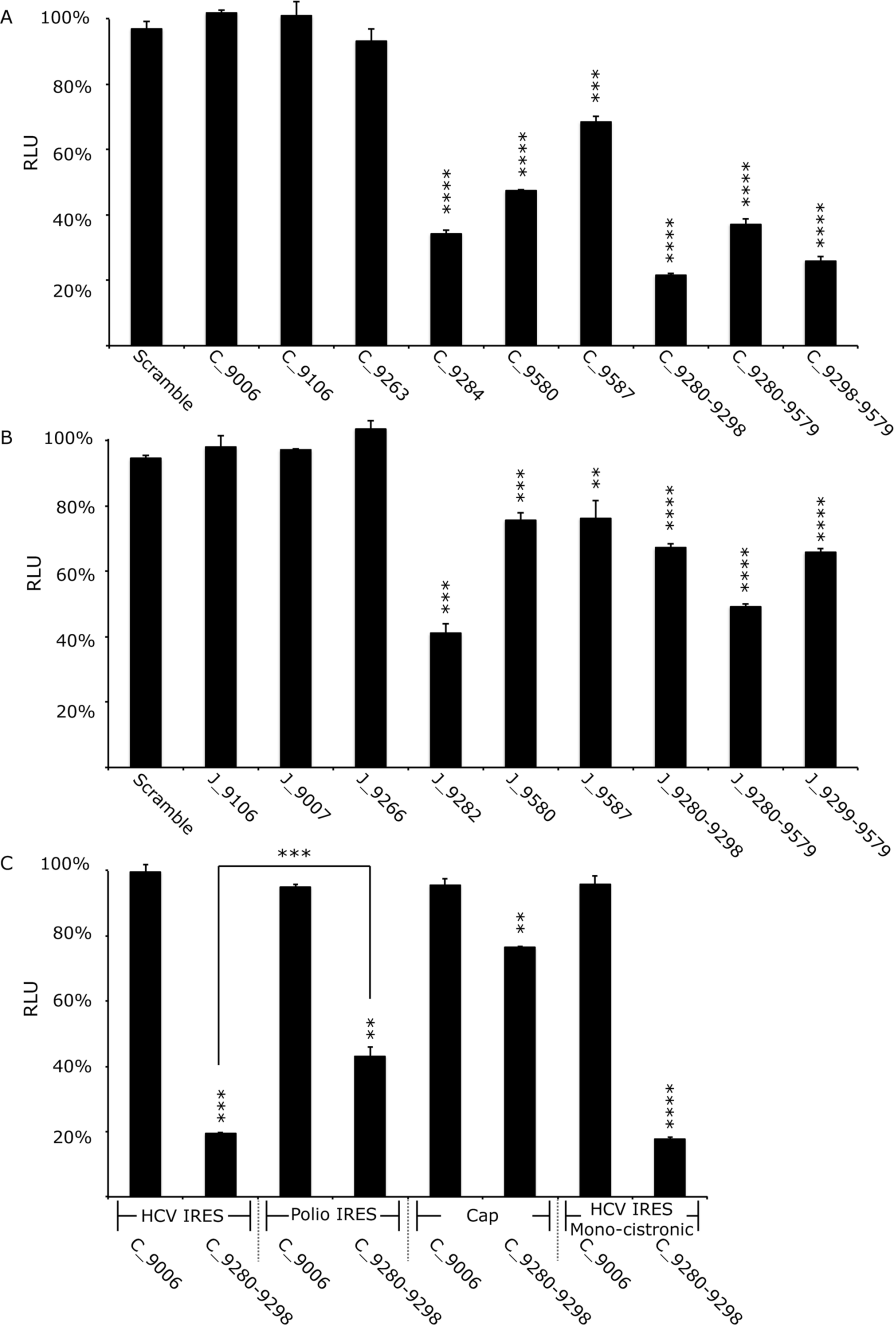
Translation in Huh 7.5 cells from Con1b (Con1b_luc_trans:ΔNS5B) and JFH-1 (JFH-1_luc_trans:ΔNS5B) translation-only reporter construct RNA in the presence of SL9266/PK antisense-LNAs or scrambled LNA-oligonucleotides. Relative luciferase levels (Firefly/Renilla) were measured 6 hours post-transfection. Results represent an average of three independent assays and are expressed as a percentage of control transfections lacking antisense-LNAs (error bars indicate standard error from the mean and stars the degrees of significance from untreated controls—or between assays connected by black lines). (**A**) Relative luciferase levels of Con1b_luc_trans:ΔNS5B RNA in the presence of antisense-LNAs. (**B**) Relative luciferase levels of JFH-1_luc_trans:ΔNS5B RNA in the presence of antisense-LNAs. (**C**) Relative luciferase levels of Con1b_luc_trans:ΔNS5B RNA in the presence of antisense-LNAs compared to variants in which the HCV 5′NCR was replaced with either a polio virus 5′NCR or m^7^G cap and a further monocistronic variant lacking the EMCV IRES and upstream NS5B region.

In order to further investigate the specific role of SL9266/PK closed and open conformations in modulating HCV IRES-initiated translation in the absence of genome replication, we developed a panel of translation-only reporter plasmids (Figure 3). We used a bacteriophage T7 polymerase-transcribed bicistronic system in which the upstream cistron was a firefly luciferase reporter gene, translation of which was driven from an HCV IRES. A second IRES—from encephalomyocarditis virus (EMCV) (36)—recruited ribosomes to translate the downstream cistron encoding the entire NS5B protein, with the RNA transcript terminating at the 3′ end of the authentic HCV 3′ NCR. Variants of this bicistronic plasmid with either Con1b- or JFH-1-derived HCV sequences were generated (Con1b_luc_trans and JFH-1_luc_trans respectively), with alternative versions deficient in NS5B translation (:ΔNS5B) through incorporation of a termination codon near the start of the ORF. Changes in levels of HCV IRES driven translation, when challenged with SL9266/PK-specific antisense-LNAs, were expressed as a function of relative luciferase expression 6 h post-transfection compared to assays lacking LNA oligonucleotides. In preliminary assays we demonstrated that NS5B translation *in cis* had no observable effect on HCV IRES-mediated translation or its inhibition, with relative luciferase expression levels indistinguishable between Con1b_luc_trans or JFH-1_luc_trans and their associated ΔNS5B versions (Supplementary data S3). Similarly, NS5B expression *in trans* had no effect on translation inhibition by SL9266/PK antisense-LNAs (data not shown). We therefore standardized on comparing luciferase reporter activity from the ΔNS5B variants of the bicistronic system (Figure 4A).

Sequence-scrambled LNA oligonucleotides or antisense-LNAs complementary to unstructured regions of the Con1b genome (LNA C_9006) had no effect on translation. Antisense-LNAs specific to domains of SL9266 involved in pseudoknot formation (C_9284 and C_9280–9298) inhibited HCV-IRES driven luciferase translation by >60% (*P* ≤ 0.0001) and >75% (*P* ≤ 0.0001) respectively. In contrast, LNAs complementary to SL9571 (C_9587 and C_9580) had a less-marked inhibitory effect of >25% (*P* ≤ 0.001) and >50% (*P* ≤ 0.0001) respectively. LNAs designed to bridge distinct domains of SL9266/PK, targeting either the terminal (C_9280-9579) or bulge loop (C_9298–9579) of SL9266 and the terminal loop of SL9571, suppressed luciferase translation from the HCV IRES by >60% (*P* ≤ 0.0001) and >70% (*P* ≤ 0.0001) respectively. As seen in the replication-based assays, antisense-LNAs targeting the 5′ side of the duplex stem of SL9266 (C_9263) or position 9110 (C_9106) had no statistically significant impact on HCV IRES driven translation. Additionally, we investigated translation and its potential inhibition in non-hepatocyte human (HeLa) or non-human (Vero) cell lines to determine whether the inhibition seen was cell-type specific (Supplementary data S4). LNA C_9284 (complementary to the single stranded loops and upper duplex of SL9266; Figure 1C) similarly inhibited translation in both cell types, though to a lesser extent than seen in Huh 7.5 cells. The differences in translation inhibition from that observed in Huh 7.5 cells were weakly significant (HeLa *P* = 0.0084 and Vero *P* = 0.0110).

The equilibrium between open and closed conformations of SL9266/PK in JFH-1 is biased towards formation of the closed structure (with the open conformation apparently favoured in genotype 1 Con1b and H77 viruses) (23). In order to determine whether this influenced HCV IRES-mediated translation we compared the luciferase signal generated from JFH-1_luc_trans:ΔNS5B and Con1b_luc_trans:ΔNS5B (Supplementary data S3). They were statistically indistinguishable, though it should be noted that – for reasons of compatibility between the IRES and SL9266/PK sequences (28)—translation was driven by different HCV IRES elements (derived from the same genotype as the NS5B/3′NCR sequences in the bicistronic vector), which may have obscured the influence of the conformation of SL9266/PK. We went on to investigate LNA-mediated inhibition of translation from bicistronic reporters containing JFH-1-derived sequences. Broadly the patterns of inhibition were similar to that observed with the Con1b-containing bicistronic system. However, although still statistically significant, relative inhibition levels were generally lower in the JFH-1 based system (Figure 4B). The greatest inhibition of the JFH-1 HCV IRES (∼60%) was observed with LNA J_9282 (*P* = 0.0004)—complementary to the single stranded loops and 3′ components of the SL9266 upper duplex—which was statistically indistinguishable from repression levels observed when targeting the same regions of SL9266/PK in Con1b_luc_trans:ΔNS5B.

In order to investigate whether inhibition of translation was specific to the HCV IRES we tested further bicistronic reporters, in which the HCV IRES of Con1b_luc_trans:ΔNS5B was replaced with the poliovirus IRES or translation was driven from an RNA in which an m^7^G ‘cap’ was added *in vitro* post-transcription (designated Polio-IRES_luc_trans and Con1b_luc_trans:Δ5′NCR respectively) (Figure 3). The effect on relative luciferase translation from these RNA templates, when the terminal and bulge loops of SL9266 were targeted with antisense-LNAs (C_9280-9298), was assayed as described earlier (Figure 4C). Translation from m^7^G 5′ capped transcripts was inhibited by ∼20% (*P* = 0.0056) and from the Polio-IRES_luc trans by ∼50% (*P* = 0.0035) (compared to >80% inhibition from the Con1b IRES parallel assays). The >30% increase in translational repression observed from the HCV IRES compared to the polio IRES was statistically significant (*P* = 0.001).

It has been suggested that translation from an upstream HCV IRES in a bicistronic system is influenced—perhaps by competition for cellular factors—by the presence of a downstream IRES (21). To investigate if this was influencing the translational repression observed in the current study we additionally constructed a monocistronic translation reporter (Figure 3). The transcript from this Con1b containing plasmid—designated Con1b_luc_trans: ΔNS5B+ΔEMCV—consisted of an upstream HCV IRES initiating firefly luciferase translation, lacked the EMCV IRES and following a stop codon incorporated the final 1452 nts of the NS5B coding region and the entire HCV 3′NCR. Following transfection of equimolar amounts of Con1b_luc_trans: ΔNS5B+ΔEMCV or Con1b_luc_trans:ΔNS5B the luciferase signals obtained were indistinguishable. Furthermore, the same level of translational repression was observed in the presence of LNA C_9280-9298 (>80%) (Figure 4C).

### Genetic analysis of the SL9266/PK interaction on translation

**Figure 5.**
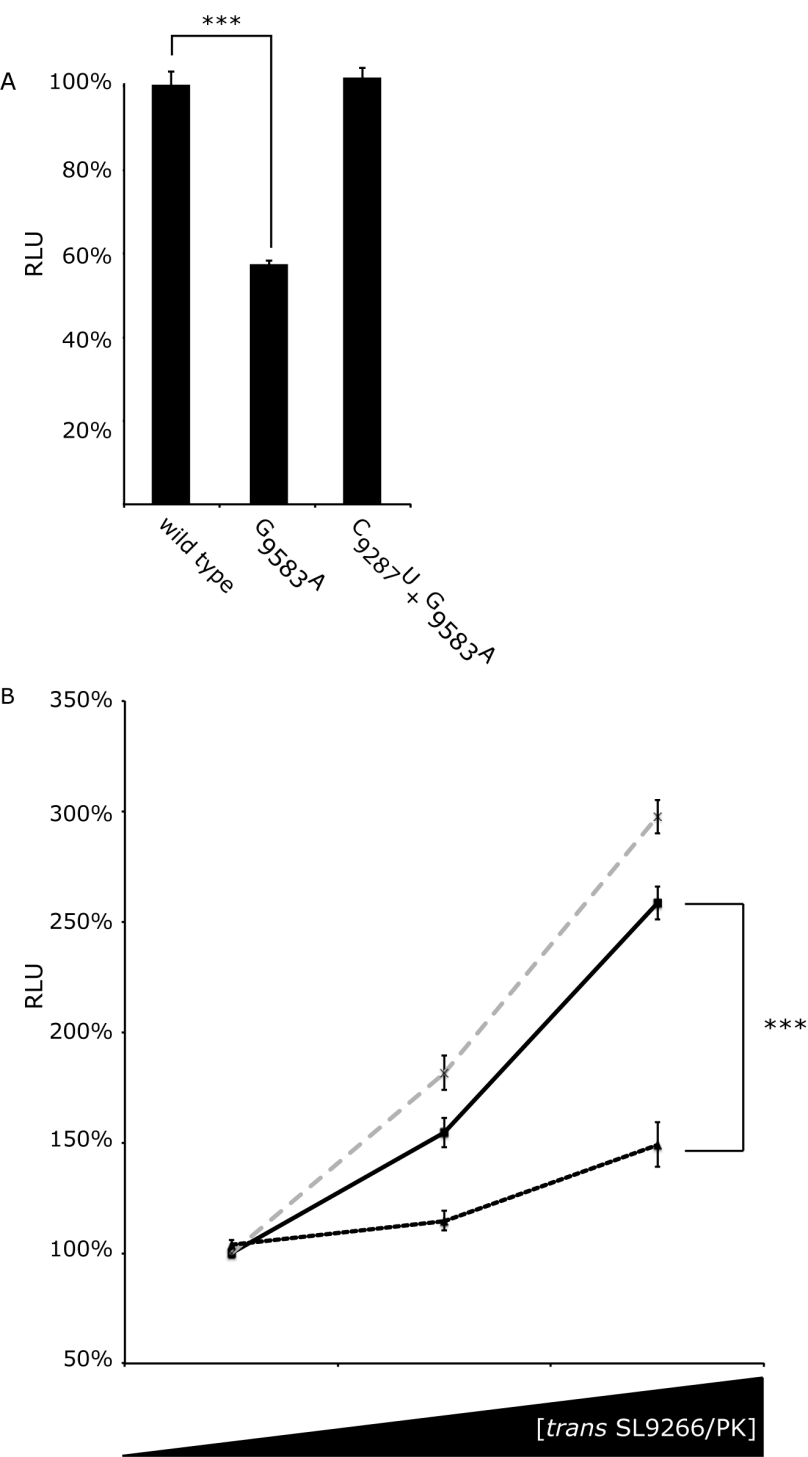
Translation in Huh 7.5 cells from SL9266/PK mutant Con1b_luc_trans:ΔNS5B RNA and the effect of adding mutant SL9266/PK RNA *in trans*. Relative luciferase levels (Firefly/*Renilla*) were measured 6 h post-transfection. Results represent the average of at least three independent assays (error bars indicate standard error from the mean and stars the degrees of significance between assays connecting black lines). (**A**) Relative luciferase levels of wild-type Con1b_luc_trans:ΔNS5B RNA compared to mutants in which the kissing loop of SL9266/PK was either destroyed (G_9583_A) or restored (C_9278_U + G_9583_A). (**B**) Relative luciferase levels of Con1b_luc_trans:ΔNS5B RNA with increasing concentrations of wild type (black unbroken line), SL9266/PK mutant G_9583_A (black dashed line) or C_9278_U + G_9583_A (grey dashed line) RNA added *in trans*. SL9266/PK *trans* concentrations increase from 0 to 1x and 5x molar excess. Results are expressed as a percentage of relative luciferase levels with no *trans* SL9266/PK RNA.

We have previously published a combination of biochemical SHAPE mapping and analysis of replication phenotypes showing that a G_9583_A substitution (in the terminal loop of SL9571) prevents formation of SL9266/PK, locking it in the open conformation and severely repressing HCV replication (∼3 log inhibition (23)). Conversely, introduction of the double mutant of C_9287_U+ G_9583_A restores both the ability of SL9266/PK to form the closed conformation and wild-type levels of genome replication. To determine the influence these modifications have on translation they were introduced into Con1b_luc_trans:ΔNS5B (Figure 5A). Translation was reduced to ∼66% of wild-type levels when the closed conformation was prevented from forming by mutation G_9583_A (*P* ≤ 0.0001) but then restored to wild-type levels by the double substitution (mutant C_9287_U+G_9583_A). Two further mutants were generated, incorporating substitutions that disrupt SL9266/PK interactions demonstrated in earlier studies (23,27) severely inhibit virus replication (by ∼3 and ∼1.5 logs respectively) through either destabilizing the upper duplex of SL9266 (A_9275_U+G_9293_U) or bulge loop RNA-RNA interactions (C_9302_A), and translation levels compared to wild-type templates. Luciferase signals were repressed by ∼20% (*P* = 0.0194) when the base-paired stem of SL9266 was disrupted and were indistinguishable from wild type when interactions of the sub-terminal bulge loop were disrupted (Supplementary data S5).

### SL9266/PK in *trans* enhances translation

We have suggested that SL9266/PK may interact with cellular or viral components during the temporal control of the virus replication cycle (23). The bicistronic assay allows quantification of translation in the absence of viral proteins. We extended this investigation to study the consequences of adding SL9266/PK *in trans* to this assay. Increasing concentrations of a non-translated NS5B-3′NCR transcript (see M&M) were co-transfected with Con1b_luc_trans:ΔNS5B before luciferase activity was quantified at 6 h post-transfection. Unmodified NS5B-3′NCR and variants bearing either G_9583_A or C_9287_U+G_9583_A substitutions were tested in parallel (Figure 5B). Wild-type SL9266/PK added in *trans* significantly stimulated translation in a dose dependent manner, with an ∼200% increase in translation at a 5x molar excess. Compared to wild-type SL9266/PK, a mutant transcript G_9583_A (locked in the open conformation) had a significantly diminished effect, stimulating translation by <50% at 5x molar excess. The extra stimulation observed for the wild-type molecule compared to mutant G_9583_A was significant at *P* = 0.0009. Wild-type levels of stimulation were restored in a double mutant C_9287_U+G_9583_A, in which ability to form the ‘kissing loop’ interaction was restored (23).

## DISCUSSION

The RNA genome of HCV is multifunctional, acting as a template for negative strand synthesis, translation of the polyprotein and a substrate for encapsidation into new viral particles. Following release into the cytosol the genome is translated by host cell machinery before negative strand synthesis generates the double-stranded replicative intermediates. In later stages of the replication cycle these are compartmentalized in membrane-associated replication complexes (RCs), presumably thereby providing functional isolation for the mutually exclusive processes of replication and translation. However, earlier in infection, before RCs are formed, temporal control is required to avoid ribosomes processing from the 5′ end of the genome clashing with the viral polymerase complex involved in negative strand synthesis (37). Where known in other virus systems, control mechanisms often function through signals at the 5′ and 3′ ends of the genome, interacting with either host cell or virally encoded factors mediated by RNA sequence and/or structure-specific binding. Such interactions may also influence genome circularization, through which the proximity of initiation and control determinants replicate and translate the genome in an ordered manner. HCV genome circularization appears to be determined, at least in part, by interaction with the poly C binding protein (PCBP2) (38). In contrast, temporal control of the replication and translation processes has yet to be fully elucidated.

SL9266, one of several phylogenetically conserved stem–loop structures in the 3′ region of the ORF, is a CRE essential for HCV replication (25,26). Structural and reverse genetic studies have shown that SL9266 takes part in various long distance RNA-RNA interactions forming pseudoknot and ‘kissing loop’ structures; disruption of which block efficient virus replication (27–30,35). Previously, we demonstrated that the critical SL9266/PK structure—formed by a ‘kissing loop’ interaction between the terminal loops of SL9266 and SL9571 within the X-tail of the 3′NCR—is dynamic and undergoes structural re-arrangement between open and closed conformations (23,31). The ability to adopt both conformations was shown as essential for efficient virus replication. We speculated that this structural rearrangement may represent an RNA switch (riboswitch), controlling or modulating HCV translation and replication. In the current study we investigated the role of SL9266/PK in specifically modulating HCV translation.

Due to the exquisite complementarity of binding, antisense-LNAs offer a means to inhibit and functionally dissect the potentially different roles of both the structure and domains of SL9266/PK; to achieve this a range of antisense-LNAs were designed and their effect on HCV replication assayed. Scrambled LNAs and those specific for non-structured regions of the genome did not inhibit virus replication. However, antisense-LNAs complementary to sequence motifs within SL9266/PK significantly inhibited replication of both the Con1b sub-genomic replicon and the J6/JFH-1 full-length virus. This concords with previous studies showing that mutations destabilizing SL9266, SL9571 or SL9266/PK inhibit virus replication and that RNA aptamers against SL9266 inhibit replication in a sub-genomic assay (39). As inhibition was observed in sub-genomic replicon systems—lacking the viral structural proteins—we can presume that SL9266/PK has a role in replication events prior to encapsidation, although additional functions cannot be excluded.

In preliminary assays, we investigated whether disruption of SL9266/PK specifically impacts HCV IRES-driven translation, using antisense-LNAs to investigate the phenotypic consequences of modulating the open or closed conformation. Relative inhibition levels of HCV IRES driven translation were indistinguishable between wild type and replication-defective (GDD>GND) Con1b sub-genomic replicons; when simultaneously targeting the terminal and bulge-loop of SL9266 or the bulge loop of SL9266 and terminal loop of SL9571 (Figure 2C). As the reporter signal generated by replication-defective mutants was reduced it strongly suggests that reduction in translation accounts for the inhibition of HCV genome replication.

To better quantify translation inhibition without the confounding issue of genome replication we developed a novel bicistronic reporter system in which luciferase expression, driven from the HCV 5′ NCR, could be tested in the presence of mutations in SL9266/PK or interacting antisense-LNAs. These studies further emphasised the importance of the ‘kissing loop’ interaction in the modulation of HCV IRES-driven translation. Antisense-LNAs complementary to the terminal loops of either SL9266 or SL9571 (or both simultaneously) significantly inhibited translation (Figure 4A and B)), thereby supporting the results obtained using replication-defective sub-genomic replicons. Previous studies have shown that mutations disrupting either the duplex-stem of SL9266 or interaction of the sub-terminal bulge loop and upstream sequences centred on nucleotide 9110 severely inhibit virus replication (at least in the Con1b system) (25,27). However, antisense-LNAs designed to interact with these regions had no influence on HCV replication (Figure 2A) or translation (Figure 4A and B). In quantified gel binding assays antisense-LNAs targeting these regions (C_9263 and C_9106) were shown to efficiently bind the template (Supplementary data S2). We propose that these apparently contradictory results may reflect LNA target inaccessibility, for example due to interaction of the translation template with host proteins, or the template adopting an inaccessible conformation in the cellular environment.

Having demonstrated that blocking or disrupting SL9266/PK inhibits HCV virus replication - at least in part - due to a specific effect on virus translation, we extended the study to examine whether this was specific to HCV IRES driven translation or was mediated through factors common to other modes of translation initiation (Figure 4C). In modified translation only reporter systems an antisense-LNA, complementary to both the terminal and bulge loop of SL9266, inhibited HCV IRES driven translation ∼2-fold more efficiently than for poliovirus IRES initiated translation and ∼4-fold more efficiently than m^7^G cap-dependent translation. The sub-terminal bulge loop of SL9266 and domain IIId of the HCV IRES include a six-nucleotide complementary motif (40). Their demonstrated interaction in *in vitro* assays has been suggested to indicate a role in modulation of translation (28,40), although we have not found evidence to support this model or interaction using SHAPE mapping and reverse genetics (23). The fact that blocking SL9266/PK significantly inhibits translation from both a m^7^G cap and poliovirus IRES - although to a lesser degree than from the HCV IRES - further suggests that SL9266/PK does not act on translation through a direct RNA–RNA interaction with the HCV IRES (the poliovirus IRES does not contain sequence motifs complementary to known interactions of the bulge-loop of SL9266 using either canonical or non-canonical pairing; data not shown). Additionally, in the present study a C_9302_A substitution in the bulge loop of SL9266 (disrupting the predicted IIId interaction) had no effect on HCV IRES driven translation, further suggesting that the SL9266-IIId interaction may not have a significant role in translation control. (Supplementary data S5). In the absence of evidence for a direct RNA–RNA interaction modulating translation we speculated that SL9266/PK could instead sequester a host *trans*-activating factor that is normally inhibitory to IRES-mediated translation.

If SL9266/PK does sequester an inhibitory cellular factor we reasoned that addition of an RNA bearing SL9266/PK *in trans* should enhance reporter gene expression. This proved to be the case, with a >2.5-fold stimulation of the HCV IRES and no effect on m^7^G cap initiated translation (Figure 5B). Furthermore, the stimulatory affect of *trans* addition of SL9266/PK in the native conformation (either unmodified or with covariant C_9287_U and G_9583_A substitutions) was ∼2-fold more effective than the open conformation (mutant G_9583_A). This suggests that it is the native conformation -a dynamic structure able to form the ‘kissing loop’ interaction - that is functionally important. It further implies that translation is stimulated by a predominantly structure-dependent mechanism, rather than being determined by the precise and highly conserved sequence at the terminal loops of SL9266 and SL9571 (30).

Here, we propose a model in which the closed conformation of SL9266/PK favours translation of the virus genome by an as-yet undetermined mechanism that may involve recruitment of one or more inhibitory cellular factors. Conversely, the open conformation - in which the ‘kissing loop’ interaction is absent and SL9571 is instead able to adopt a stem–loop structure (23) - favours replication (41). Interestingly, the cellular protein EWSR1 has recently been shown to enhance HCV replication through preferential interaction with SL9266 in the open conformation (41). In a model proposed by Oakland *et al*. (41), the switch from translation to replication involved disruption of the ‘kissing loop’ interaction, favouring recruitment of EWSR1 and initiation of replication. What remains unclear from these related models is whether a feedback mechanism exists that represses translation - for example, after sufficient non-structural proteins have been synthesised - to enable genome replication.

**Figure 6.**
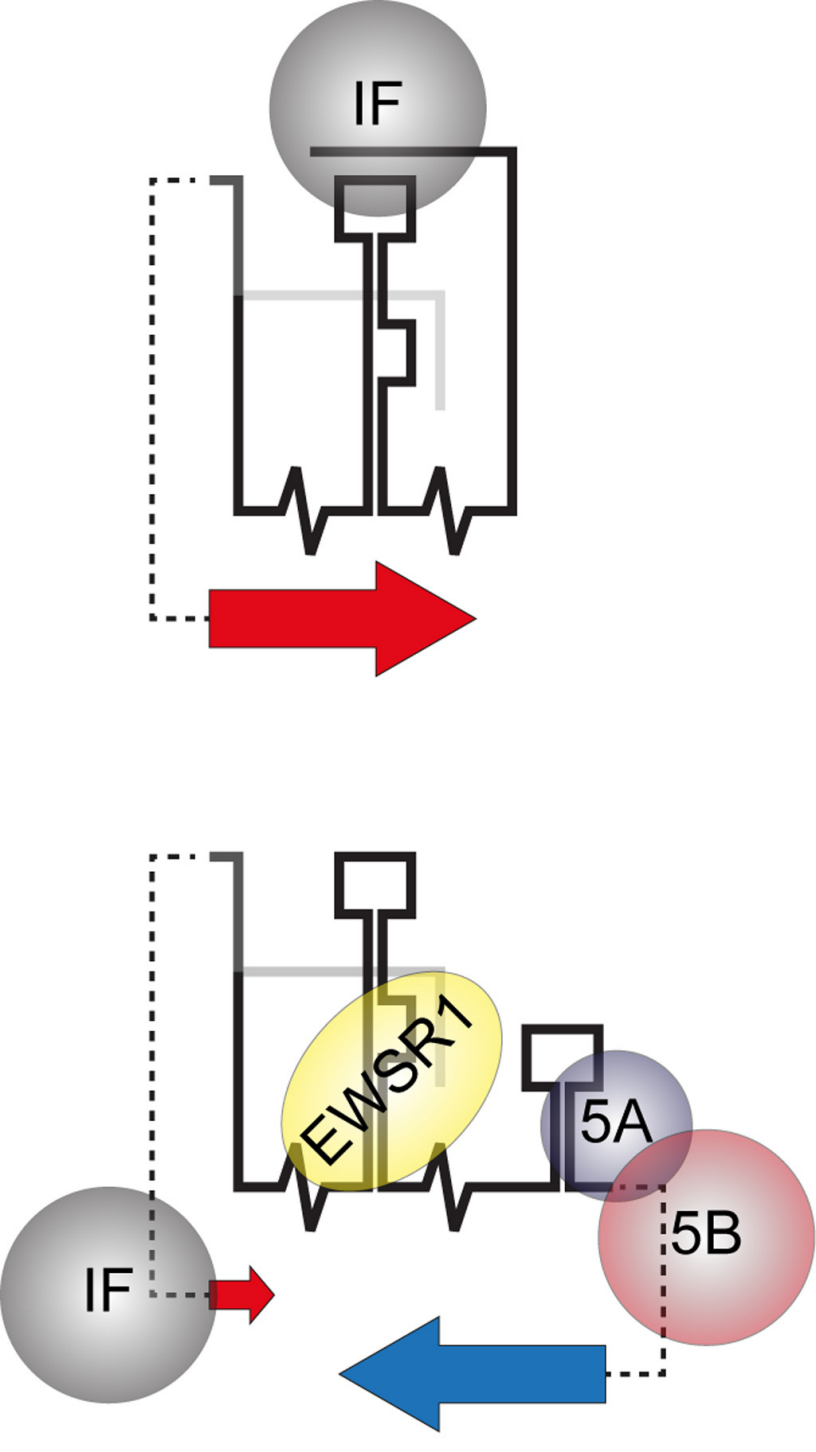
A schematic interpretation of SL9266/PK conformational rearrangement as part of a translation/replication switch; illustrating the mechanism and role of negative feedback interactions in the proposed model. The upper schematic illustrates up-regulation of HCV IRES initiated translation (large red arrow) due to the SL9266/PK closed conformation sequestering a cellular IRES inhibition factor (IF). The lower cartoon illustrates the open conformation, initiated by virally encoded non-structural proteins (5A and 5B) binding the 3′NCR and blocking ‘kissing loop’ formation. The open structure favours interaction between cellular protein EWSR1 and SL9266, up-regulating RNA replication (large blue arrow) (41). As demonstrated in the current study the resulting open conformation removes up-regulation of HCV translation (small red arrow), which we speculate is due to EWRS1 displacing - and thus making available - translation inhibition factor IF.

We speculate that such a feedback mechanism may function through the interaction of SL9266/PK with virally encoded non-structural proteins. Non-structural proteins NS5A - critical for efficient virus replication (42,43) - and NS5B both bind the virus 3′UTR (44,45); NS5A to the poly U/UC tract (46) and NS5B to the X-tail (46). They have also been shown to interact with each other, modulating negative-strand synthesis (43,47,48). We hypothesize that by binding in the region of SL9571, NS5A and 5B initiate a negative feedback to HCV translation by blocking formation of the ‘kissing loop’, resulting in stabilization of SL9266/PK in the open-conformation thus favouring binding of EWSR1 to SL9266 and consequently up-regulating genome replication (Figure 6). In the current study, expression of NS5B *in cis* had no affect on HCV translation or antisense-LNA inhibition (Supplementary data S3). This is consistent with the suggestion that both NS5A and 5B may be required, acting in concert to stimulate replication (46). A logical extension of this model would be that their recruitment inhibits the interaction of SL9266/PK with the proposed translational inhibitory factor. A consequence of this would be translation suppression at the same time as genome replication is initiated (Figure 6).

The relationship between the structural complexity and multi-functional nature of the RNA genome of HCV is gradually being elucidated. The studies presented here propose a model within which the temporal control of replication events and the contribution made by viral and cellular proteins can be investigated. These studies are important both for our understanding of HCV and related viruses and, since they are likely to be evolutionary conserved features in what are otherwise highly variable viruses, provide potential targets for therapeutic intervention.

## SUPPLEMENTARY DATA

Supplementary Data are available at NAR Online.

SUPPLEMENTARY DATA
