# Supplementary material for: Inhibition of HCV translation by disrupting the structure and interactions of the viral CRE and 3′ X-tail
Source: Nucleic Acids Res. 2015 Feb 20;43(5):2914–26. doi: 10.1093/nar/gkv142 (PMC4357731; doi:10.1093/nar/gkv142)
Supplement: SUPPLEMENTARY DATA [file supp_43_5_2914__index.html]

Inhibition of HCV translation by disrupting the structure and interactions of the viral CRE and 3′ X-tail — SUPPLEMENTARY DATA 

# Inhibition of HCV translation by disrupting the structure and interactions of the viral CRE and 3′ X-tail

## SUPPLEMENTARY DATA

**Files in this Data Supplement:**

- SUPPLEMENTARY DATA
