# Supplementary material for: Inhibition of HCV translation by disrupting the structure and interactions of the viral CRE and 3′ X-tail
Source: Nucleic Acids Res. 2015 Feb 20;43(5):2914–26. doi: 10.1093/nar/gkv142 (PMC4357731; doi:10.1093/nar/gkv142)
Supplement: SUPPLEMENTARY DATA [file supp_gkv142_nar-03661-r-2014-File009.pdf]

Antisense LNA-oligonucleotide name	Sequence
C_9284	+G+G+GC+ACGAG+ACAG+GCTGT+G+A+T
C_9263	+T+G+TCTCC+CC+CGC+TG+T+A+A
C_9587	+G+G+CTC+A+CGG+AC+C+T+T
C_9580	+T+C+AACGG+ACC+TTT+CAC+A+G+C
C_9280-9298	+T+C+G+GGCA+CTCTAA+GCTG+TGATA+T+A
C_9298-9579	+T+T+TCACAG+CTTCTT+CG+GG+C+A+C
C_9280-9579	+T+T+TCgCgG+CTTTTC+TGTGA+T+A+T+A
C_9106	+G+G+ACA+CTTC+TGGCC+C+G+A+T
J_9282	+G+G+GCGCGCG+AC+ACGCT+GTG+A+A+A
J_9266	+T+G+TCG+CC+C+CCG+C+C+G
J_9587	+G+G+CTC+A+CGG+AC+C+T+T
J_9580	+T+C+AACGG+ACC+TTT+CAC+A+G+C
J_9280-9298	+T+C+GGGCG+CTAATT+GCT+GTGAA+A+A+A
J_9299-9579	+T+T+TCA+CAGC+TTCTT+CGG+G+C+G
J_9280-9579	T+T+TCgCgG+CTTTTTTC+TG+TGgg+g+g+g
J_9106	+T+G+AC+TGC+GCG+AGCCC+G+A+C
J_9007	+G+C+ATA+GAA+AAG+GCG+T+C+A

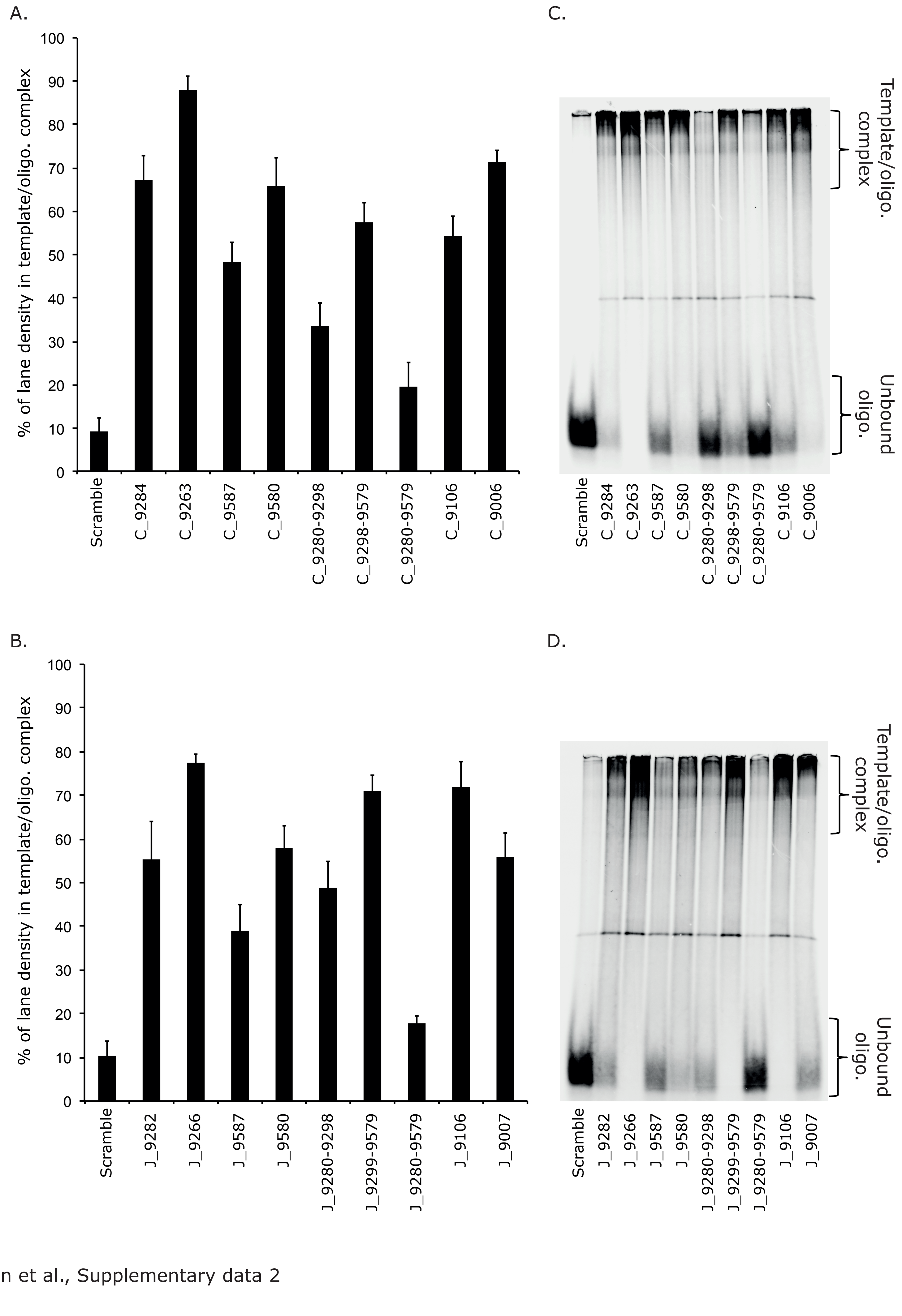

Tuplin et al., Supplementary data 2

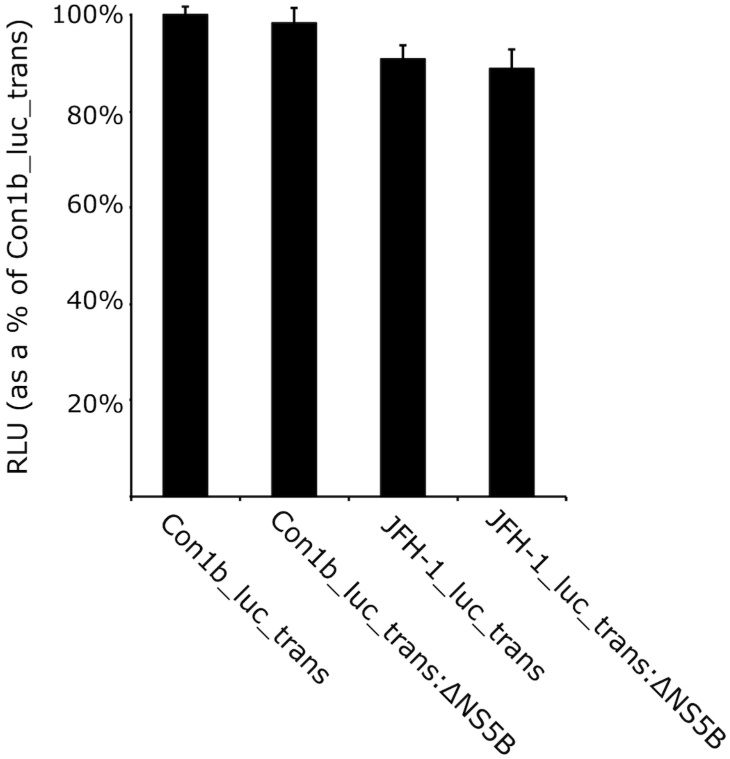

Tuplin et al., Supplementary data 3

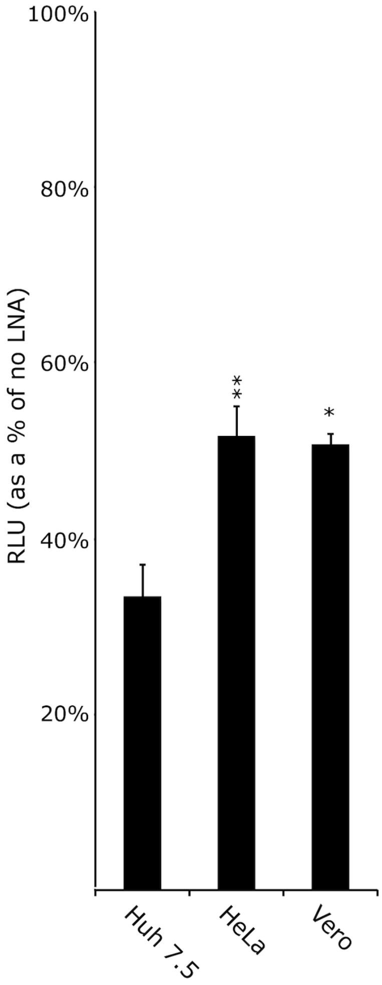

Tuplin et al., Supplementary data 4

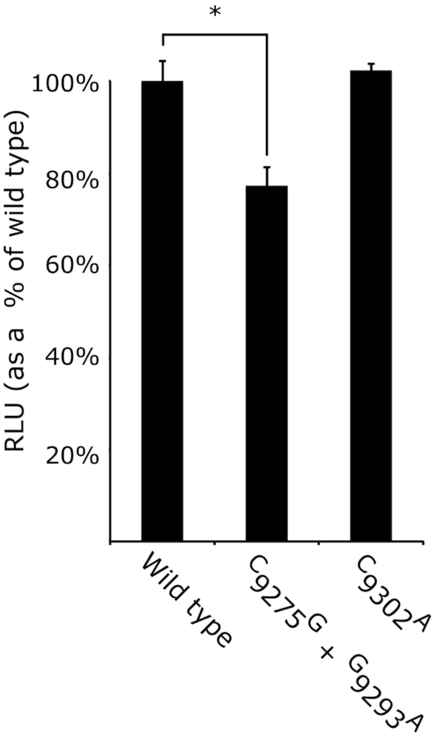
*
100%
80%
60%
40%
20%
RLU (as a % of wild type)
Wild type
C9275G + G9293A
C9302A

Supplementary data 1

Sequence of antisense oligonucleotides.

LNA nucleotides indicated by a proceeding +, non-complementary linker sequences are underlined and complementary non-canonical nucleotides by lowercase letters.

Supplementary data 2

Gel shift assay quantification showing antisense-LNA oligonucleotides / RNA template binding efficiencies

Equimolar ratios of radiolabeled (^{33}P-5′) LNA-oligonucleotides and either Con1b **(A)** or JFH-1 **(B)** full length RNA template were combined and incubated at 37°C for 20 min before separation on 7% native PAGE gels. Following phosphor screen exposure, binding efficiencies were quantified by densitometry and expressed as relative levels of shifted template/LNA-oligonucleotide complex as a percentage of total lane density for a non-specific scrambled control and each antisense LNA-oligonucleotide. Representative gel shift images for Con1b **(C)** and JFH-1 **(D)** are shown adjacent to corresponding quantification. Results represent an average of three independent assays and error bars indicate the standard error from the mean.

Supplementary data 3

Translation from Con1b and JFH-1 IRESs compared with and without NS5B expression in cis

Translation in Huh 7.5 cells from Con1b_luc_trans, Con1b_luc_trans:ΔNS5B, JFH-1_luc_trans and JFH-1_luc_trans:ΔNS5B. Relative luciferase levels (Firefly/Renilla) were measured 6 hours post-transfection and expressed as a percentage of Con1b_luc_trans. Results represent the average of at least three independent assays and error bars indicate the standard error from the mean.

Supplementary data 4

SL9266/PK antisense-LNA (LNA C_9280-9298) inhibition of translation from Con1b_luc_trans:ΔNS5B RNA across different cell types. Relative luciferase levels (Firefly/Renilla) were measured 6 hours post-transfection. Results represent the average of three independent assays and are expressed as a percentage of control transfections lacking antisense-LNAs (error bars indicate the standard error from the mean and stars the degrees of significance from Huh 7.5 results).

Supplementary data 5

Translation in Huh 7.5 cells from SL9266/PK mutant Con1b_luc_trans:ΔNS5B RNA. Relative luciferase levels (Firefly/Renilla)

were measured 6 hours post-transfection. Results represent the average of at least three independent assays as a percentage of wild type RNA translation (error bars indicate the standard error from the mean and stars the degrees of significance between assays connected by a black line).
